# Trends in antibiotic prescribing in primary care out-of-hours doctors’ services in Ireland

**DOI:** 10.1093/jacamr/dlae009

**Published:** 2024-02-09

**Authors:** Mala Shah, Teresa M Barbosa, Gary Stack, Aoife Fleming

**Affiliations:** Community Operations, Health Service Executive, Cork, Ireland; Pharmaceutical Care Research Group, School of Pharmacy, University College Cork, Cork, Ireland; Pharmaceutical Care Research Group, School of Pharmacy, University College Cork, Cork, Ireland; SouthDoc Out of Hours Doctors Service, Cork and Kerry, Ireland; Pharmaceutical Care Research Group, School of Pharmacy, University College Cork, Cork, Ireland; Pharmacy Department, Mercy University Hospital, Grenville Place, Cork, Ireland

## Abstract

**Background:**

Infections are a common reason for patient consultation in out-of-hours (OOH) doctors’ services. Surveillance of antibiotic prescribing in OOH settings is important to develop tailored antimicrobial stewardship (AMS) interventions.

**Objectives:**

To evaluate antibiotic prescribing patterns in OOH services in the Cork Kerry region, Ireland to inform future AMS interventions.

**Methods:**

A retrospective, observational cohort study was conducted of all oral antibiotic prescriptions in OOH doctors’ consultations between 1 December 2019 and 31 December 2021 in the region. Data were gathered on age, gender, date and time of consultation, consultation method (in person, remote), antibiotic and its indication. Data were analysed using Microsoft Excel v.2018 and SPSS v.28.

**Results:**

Overall, 17% (69 017 of 406 812) of the OOH doctors’ consultations resulted in an antibiotic prescription during the study period. This varied from 31% of OOH consultations in December 2019 to less than 2% of OOH consultations in April 2020. Of the antibiotics prescribed, 21% were for children under 6 years old. Respiratory tract infections (RTIs) were the most common indication for antibiotics (59%). Amoxicillin was the most commonly prescribed antibiotic (40% of all prescriptions). Red (reserved) antibiotics accounted for 19% of all prescriptions. During the COVID-19 pandemic period of the study, 66% of 49 421 of antibiotic prescriptions were issued from remote consultations.

**Conclusions:**

Low antibiotic prescribing levels during the early stages of the pandemic were not sustained. Antibiotic prescriptions from remote consultations were common. A key opportunity for AMS is addressing the volume of antibiotic prescribing for RTIs, particularly in children.

## Introduction

Antibiotics are a lifesaving intervention and play a significant supporting role in medical advances. However, antibiotic use also drives antibiotic resistance,^1^ which is a major threat to public health. In Ireland, antimicrobial consumption in primary care is higher than the European mean.^2^ Investigating antibiotic prescribing patterns to identify where improvements can be made is important to plan (AMS) antimicrobial stewardship interventions to ensure antibiotics are used wisely to preserve their usefulness for future generations.^3,4^

Out-of-hours (OOH) doctors services provide patient access to general practitioners (GPs) for urgent medical needs in primary care outside of normal daytime practice hours. Infections are a common reason for OOH presentations, as described in a study from the Netherlands where infections accounted for 26% of OOH consultations.^5^ The prevalence of antibiotic prescribing in OOH services has been reported as 15% of all OOH doctor consultations in studies conducted in the UK, Denmark and Norway^6–8^, and 33% in Iceland.^9^ Antibiotics prescribed in the OOH setting account for 5% to 6% of the volume of overall antibiotic consumption in primary care in the UK and the Netherlands.^10,11^

Compared to in-hours GP services, a higher proportion of patients visiting OOH services are prescribed an antibiotic.^10–12^ In Ireland, one study reported 60% of children presenting with upper respiratory tract infections (RTIs) in an OOH service were prescribed an antibiotic.^13^ OOH consultations are more likely to be for acute presentations that cannot safely wait until the patients’ own GP surgery re-opens. Unfamiliarity between doctor and patient, patient medical history and laboratory results not being available, limited access to diagnostics and lack of follow up on patient progress are additional challenges in the OOH setting that influence higher antibiotic prescribing rates. High workload and time constraints in OOH settings have also been reported to increase the likelihood of antibiotic prescribing.^14,15^

Although antibiotic use surveillance is an important component in antibiotic stewardship (AMS),^3,16^ there is no centralized data collection of antibiotic prescriptions in Ireland in the OOH setting. A quality improvement initiative conducted in 2017–2018 in several treatment centres in the Cork Kerry region OOH services introduced an antibiotic trigger tool to nudge prescribers to choose preferred antibiotics for empiric treatment of infection.^17^ Following the success of this initiative, the antibiotic trigger tool was implemented in all treatment centres in the region. This has enabled recording of data on antibiotics in the Cork Kerry region OOH service since December 2019. A detailed descriptive analysis of antibiotic prescribing in the OOH setting has not previously been undertaken in Ireland. This study aimed to describe the patterns of antibiotic use in the Cork and Kerry region OOH doctors services from 2019 to 2021 to identify opportunities for AMS, which are also likely to be applicable to OOH services in other regions.

## Methods

### Ethics

Ethical approval was obtained from the Clinical Research Ethics Committee of the Cork Teaching Hospitals. Reference number ECM 4 (s) 11/01/2022.

### Design and setting

To describe the frequency and characteristics of antibiotic prescribing in the Cork Kerry region OOH doctors’ services, a retrospective, observational cohort study was conducted of all oral antibiotic prescriptions in OOH doctors’ consultations between 1 December 2019 and 31 December 2021. The Cork and Kerry region has a population of approximately 690 575.^18^

Daytime GP practice hours can vary, and are usually between 8am and 6pm. Outside normal daytime general practice hours, for urgent medical needs that cannot wait until the following working day, OOH doctors services are available at 23 treatment centres across the Cork Kerry region. When patients contact the service, they are triaged by a team of nurses who decide the level of healthcare need and whether a doctor consultation is required. The doctor consultations are conducted either in person at the treatment centre or a home visit, and since the COVID-19 pandemic telephone consultations also take place. A cooperative of approximately 500 GPs who also work in daytime GP practices cover the ‘bright-eye’ OOH sessions during evenings (6pm to 10pm), weekends and bank holidays (8am to 10pm), and a set of locum doctors who generally do not provide daytime GP care, cover overnight ‘red-eye’ shifts (10pm to 8am). Point of care diagnostics are not available in this OOH service and a decision to prescribe antibiotics is generally made on clinical presentation, as in most daytime GP practices.

Similar to the WHO AWaRe antibiotic classification,^19^ in Ireland, a preferred antibiotic initiative for community settings lists antibiotics used in primary care as either Green (preferred) or Red (reserved) agents.^20^ Green agents are advocated for empiric treatment of commonly encountered infections, as they are likely to be effective, have fewer adverse effects and are less likely to contribute to antibiotic-resistant infections. Green antibiotics include phenoxymethylpenicillin, amoxicillin, doxycycline, flucloxacillin, nitrofurantoin, trimethoprim, cefalexin and fosfomycin. Red antibiotics include co-amoxiclav, other cephalosporins, macrolides, quinolones and clindamycin. A notable difference from the WHO AWaRe (Access, Watch, Reserve) classification is that co-amoxiclav is classed as an ‘Access’ (similar to Green) antibiotic by the WHO but is a Red (reserve) antibiotic in Ireland.

### Data and data analysis

The following information was available for all antibiotic prescriptions: date and time prescribed, age of patient, gender, time of consultation, consultation method (in person, telephone, home visit) and antibiotic prescribed. Although the presenting problem was not available for the OOH consultations, the indication for the antibiotic was available for 77% of data entries as free text. The total number of OOH doctors’ consultations per month for the previously mentioned time period was available.

Using Microsoft Excel v.2018, data was categorized as follows: time period (month/year), gender of patient (male or female), age of patient (0 to 6 years, 7 to 16 years, 17 to 64 years or 65 years and over), method of consultations (remote or in person) and shift type (bright-eye or red-eye). Antibiotics were categorized as Green or Red agents as per the national preferred antibiotic initiative. Metronidazole was categorized as a Green antibiotic for the purpose of this study (not categorized in the national preferred antibiotic list).

The free text information on the indication for the antibiotic was categorized by anatomical site of infection: RTI, urinary tract infection (UTI), skin or soft tissue infection (SSTI), dental infection and ‘other’, which included sexually transmitted infections (STI), pregnancy and post-partum infections and intra-abdominal infections.

Assessment of antibiotic choice was made against the first-choice agents recommended in the national antimicrobial guidelines for community setting available at www.antibioticprescribing.ie.

Data analysis was conducted using SPSS v.28. A descriptive analysis of antibiotic prescription frequencies was conducted and the proportion of Red antibiotics per gender, age category, shift type, method of consultation and infection type was determined. Chi-squared tests were conducted to investigate the difference between the proportion of Red antibiotics prescribed per shift type and gender and the proportion of antibiotics prescribed in remote versus in-person consultations.

Analysis of remote versus in-person antibiotic prescriptions was conducted for data from 1 April 2020 to 31 December 2021, as before this time, prescribing of antibiotics in remote consultations rarely occurred.

Population level data for the Cork and Kerry region was taken from the 2016 National Census^17^ to calculate number of OOH consultations per 1000 inhabitants per year. The number of OOH antibiotic prescriptions per 1000 inhabitants per year for 0–6-year-olds and 65-year-olds and over was calculated as data on these age categories were readily available from the census data.

## Results

### Number of OOH consultations and antibiotic prescriptions

Between December 2019 and December 2021, there were 406 812 doctor consultations in the Cork and Kerry region, 17% (69 017) of which resulted in an antibiotic prescription. There were 260 OOH doctor consultations per 1000 inhabitants in 2020 and 286 per 1000 inhabitants in 2021. There were 37 antibiotic prescriptions per 1000 inhabitants per year in 2020 and 50 per 1000 inhabitants per year in 2021. For age 0–6 years, there were 65 antibiotic prescriptions per 1000 children in 2020, rising to 113 per 1000 in 2021 (Table S1, available as Supplementary data at *JAC-AMR* Online).

**Figure 1.**
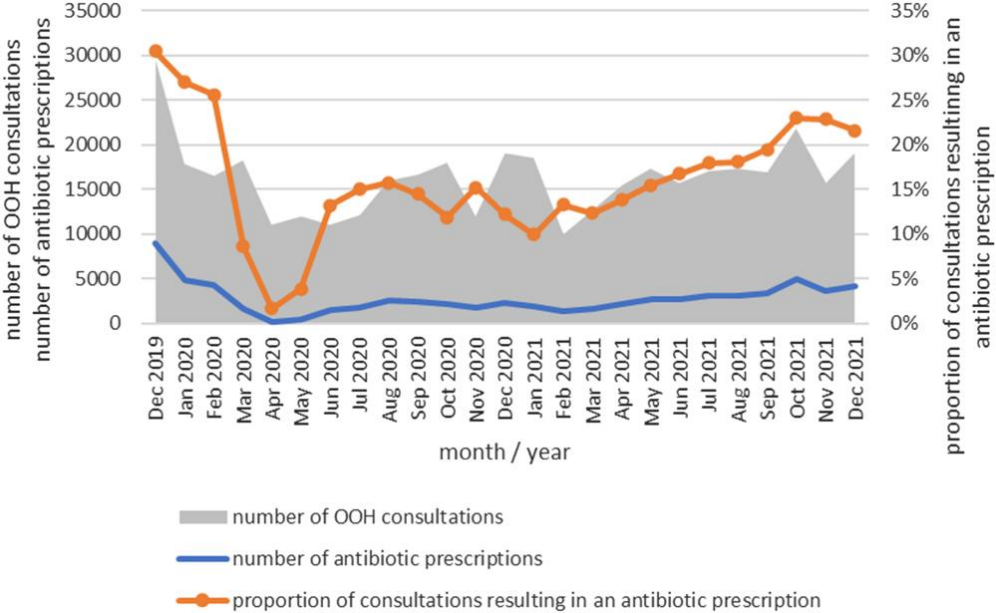
Trends in number of OOH consultations, antibiotic prescriptions and proportion of consultations resulting in an antibiotic prescription.

The total number of OOH consultations was highest in the month of December 2019 (29 490) and lowest in April 2020 (10 999). Over the study period, the proportion of OOH consultations resulting in an antibiotic prescription ranged from a peak of 31% (8998/29 490) in December 2019, to less than 2% (183/10 999) in April 2020 at the start of the COVID-19 pandemic (Figure 1). From June 2020 to May 2021, the proportion of consultations resulting in an antibiotic prescription ranged from 10% to 15%. From March 2021 there was a steady increase in the proportion of consultations resulting in an antibiotic prescription, which peaked at 23% (5017/21 815) in October 2021.

### Antibiotic prescription characteristics

**Figure 2.**
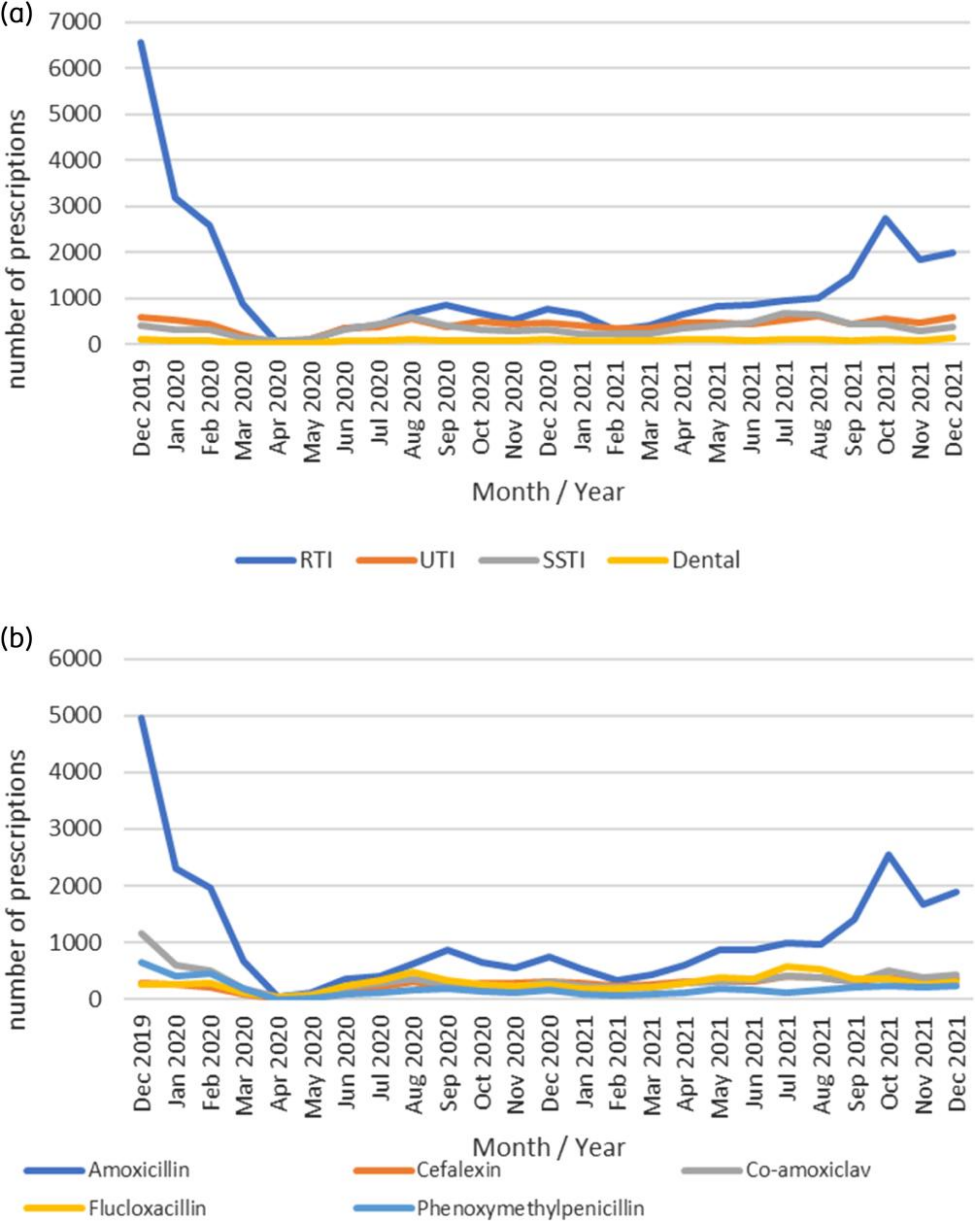
Trends in indication for antibiotics (a) and top five antibiotics over time (b).

The median age for patients receiving antibiotic prescriptions was 29 years (IQR 8–51yrs). Table 1 illustrates the number and proportion of total antibiotic prescriptions and Red antibiotics per gender, age category, shift type and indication. Of the 69 017 antibiotics prescribed, 60% (*n* = 41 244) were for females and 21% (*n* = 14 148) were for children 0–6 years old. RTIs were the most commonly recorded indication for antibiotics (59%, *n* = 31 174/53 211) of all antibiotic prescriptions where indication was recorded, followed by UTIs (20%, *n* = 10 546/53 211) and SSTIs (16%, *n* = 8667/53 211). The greatest variation in prescribing by indication was seen in antibiotic prescriptions for RTIs, with the largest peak in December 2019 and then in October 2021 (Figure 2). The number of antibiotic prescriptions for most other infection types remained stable over the study period. There were some notable differences between the number of antibiotics prescribed for different indications categorized by age group. RTIs were the predominant infection type treated in children (70%), while the proportion of antibiotic prescriptions for UTIs increased with each age category, ranging from 5% in the 0–6 years group to 31% in the 65 years and over group (Table S2). Some differences in antibiotics prescribed by gender were also noted (Table S3). Males accounted for a higher proportion of antibiotics for STIs (95%), and females for UTIs (82%).

**Table 1.** Breakdown of overall antibiotic and Red antibiotic use

	Total antibiotic prescriptions		Red^a^ antibiotic prescriptions	
	*n*	%	*n*	percentage of total antibiotics (%)
**All**	69 017	100	13 117	19.0
**Gender**				
Female	41 244	59.7	7321	17.8
Male	27 793	40.3	5796	20.9^b^
**Shift type**				
Bright-eye	58 928	85.4	10 344	17.6
Overnight	10 089	14.6	2773	27.5^b^
**Age category** ^c^				
0–6 years	14 148	20.6	2096	14.8
7–16 years	8901	12.9	1576	17.7
17–64 years	35 005	50.9	6909	19.7
65 years +	10 714	15.6	2518	23.5
**Indication** ^d^				
RTI	31 174	58.6	5487	17.6
UTI	10 546	19.8	1756	16.7
SSTI	8667	16.3	1923	22.2
Dental	2001	3.8	466	23.2
Other	823	1.5	430	52.2

^a^Examples for Red antibiotics include co-amoxiclav, macrolides, quinolones, clindamycin and cephalosporins (excluding cefalexin).

^b^Chi squared test significant at *P* < 0.05.

^c^Age was available for 68 768 of 69 017 prescriptions.

^d^Indication was known for 77% (*n* = 53 211/69 017) prescriptions.

Weekends accounted for 59% (40 956/69 017) of all antibiotic prescriptions and, overall, during both weekdays and weekends, most (85%, *n* = 58 928) antibiotics were prescribed during the bright-eye shift.

Before April 2020 in-person doctors’ consultations represented 86% of all consultations. From 1 April 2020 to 31 December 2021, remote consultations accounted for 75% of OOH doctors consultations, reflecting the shift in consultation type due to the COVID-19 pandemic. Before April 2020, antibiotics were rarely prescribed in remote consultations. However, since April 2020, remote consultations accounted for 66% (32 388/49 421) of all antibiotic prescriptions from 1 April 2020 to December 2021, and throughout this period, accounted for a higher proportion of antibiotic prescriptions than in-person consultations (Figure S1). A lower proportion of Red antibiotics was prescribed in remote consultations [18% remote (5847/32 388) versus 23% in person (3946/17 033), chi-squared test *P* < 0.05] during the same time period.

### Antibiotic choice

The most frequently prescribed antibiotic was amoxicillin (40% of all antibiotic prescriptions), followed by co-amoxiclav (12%), flucloxacillin (10%), cefalexin (10%) and phenoxymethylpenicillin (7%). Quinolones accounted for less than 2% of antibiotic prescriptions (Table S4). Trends of the number of antibiotic prescriptions for the five most commonly prescribed agents per month show a marked increase in the number of amoxicillin prescriptions in December 2019 and October to December 2021 while the number of prescriptions per month for most other antibiotics remained relatively stable over the course of the study period (Figure 2).

Amoxicillin was the most commonly prescribed agent for RTIs and dental infections, cefalexin for UTIs and flucloxacillin for SSTIs (Table 2).

**Table 2.** Most commonly prescribed antibiotics for main infection types

Respiratory tract infection *n = 31 174*			Urinary tract infection *n = 10 546*			Skin infection *n = 8667*			Dental infection *n = 2001*		
antibiotic	*n*	%	antibiotic	*n*	%	antibiotic	*n*	%	antibiotic	*n*	%
amoxicillin	19 027	61.0	cefalexin	4559	43.2	flucloxacillin	5076	58.6	amoxicillin	1167	58.3
co-amoxiclav	3219	10.3	nitrofurantoin	1924	18.2	co-amoxiclav	1528	17.6	co-amoxiclav	392	19.6
penicillin V	3075	9.9	trimethoprim	1600	15.2	doxycycline	515	5.9	metronidazole	207	10.3
clarithromycin	2075	6.7	co-amoxiclav	1105	10.5	penicillin V	263	3.0	clarithromycin	57	2.8
doxycycline	1916	6.1	amoxicillin	512	4.9	clarithromycin	256	3.0	penicillin V	53	2.6

Assessment of prescribing against the following guideline-recommended agents showed that for RTIs amoxicillin, phenoxymethylpenicillin and doxycycline accounted for 77% of antibiotic prescriptions. For UTIs cefalexin, nitrofurantoin and trimethoprim accounted for 77% of antibiotic prescriptions. For SSTIs, flucloxacillin accounted for 59% of all prescriptions. For dental prescriptions, amoxicillin and metronidazole accounted for 69% of the prescriptions. Co-amoxiclav, a Red (reserve) antibiotic, accounted for 10% of the RTIs prescriptions, 18% of SSTIs and 20% of dental antibiotic prescriptions.

## Discussion

This study describes the patterns of antibiotic prescribing in the Cork Kerry region OOH services during a period of profound change when patient consultations to OOH services were affected by the COVID-19 pandemic. Antibiotics were prescribed on average in 17% of OOH doctor consultations over the study period, however, there was a wide variation in the proportion of consultations resulting in an antibiotic prescription, with a substantial decrease at the start of the pandemic, increasing in 2021. There was a notable increase in antibiotic prescriptions for young children in 2021. RTIs were the main indication for antibiotics.

### Antibiotic prescribing trends and comparison to existing literature

December 2019 saw the highest number of consultations and the highest proportion of consultations resulting in an antibiotic prescription in this OOH service. This coincided with an early influenza season,^21^ which often correlates to increases in antibiotic consumption in primary care.^22^ A sharp decrease in OOH antibiotic prescriptions in the early stages of the pandemic was also reflected in the national antimicrobial consumption data for primary care in Ireland^23^ and internationally in studies conducted in OOH settings.^12,24,25^ There was no winter peak in antibiotic prescriptions in 2020–2021, at a time of further restrictions and school closures. From March 2021, there was an increase in the proportion of OOH antibiotic prescriptions, especially evident in the 0–6-year age group, coinciding with the easing of COVID-19 restrictions nationally and greater potential for transmission of RTIs.

OOH doctor consultation rates per population in this study (260 per 1000 residents in 2020) were slightly higher than reported in a study conducted in the Netherlands during the same time period (231 per 1000 inhabitants, 2020).^26^ The study OOH consultation rates are considerably higher than those reported in studies conducted before the pandemic: Belgium (63 per 1000 inhabitants, 2016), Netherlands (145 per 1000 inhabitants, 2016) and Denmark (80 per 1000 inhabitants, 2011).^27,28^ It is likely that OOH consultation rates, and therefore antibiotic prescription rates, were affected by the COVID-19 pandemic. In Ireland, in 2020, doctors were the only referral pathway for COVID-19 PCR testing, which was the only recommended test for COVID-19 in Ireland, and this is likely to have resulted in a higher OOH doctor consultation rate. Varying organizational structures of primary care health services in different countries may also affect OOH consultation rates.

Although the overall proportion of OOH consultations resulting in an antibiotic prescription over the study period (17%) is similar to 15% reported from three OOH antibiotic prescribing studies from the UK, Denmark and Norway,^6–8^ those studies were conducted prior to the COVID-19 pandemic, therefore direct comparison to this study period may not be meaningful. A Dutch study conducted over the pandemic period observed a lower proportion of consultations resulting in an antibiotic prescription (6%–10%).^12^ However, in the Netherlands antibiotic consumption in primary care in general is also lower per head of population than in Ireland.^2^ Regional or national figures for in-hours doctor consultations resulting in an antibiotic prescription in Ireland are not available for the study period. A study conducted in 2008–2010 reported 20% consultations in Irish general practice resulted in an antibiotic prescription,^29^ but no corresponding data for OOH consultations is reported for that time period.

The median age of patients prescribed antibiotics (29 years) is similar to other OOH studies,^6,9^ and lower than the average national age (37 years).^18^ Females and young children were prescribed a higher proportion of antibiotics, similar to observations from UK, Denmark and Iceland OOH studies.^6,7,9^ The higher proportion of females prescribed antibiotics is likely to be a result of higher rates of consultations in women as described in an English study of GP consultations for infections.^30^ Findings from our study indicating a higher proportion of males prescribed antibiotics for STIs, and for females prescribed antibiotics for UTIs offers areas for targeted education for patients on preventative measures.

A lower threshold for parents seeking OOH doctor consultations in children^31^ and high prescribing rates in children^32^ have been described in other European countries. In Ireland, since the introduction of the free doctor consultations for children under 6 years in 2015, there has been an increase in the number of presentations to the OOH service in this age category, affecting the quantity of antibiotics prescribed. The high rate of antibiotic prescribing in young children presents a challenge and opportunity for AMS. Studies highlight challenges with parent expectation of antibiotics,^33^ greater satisfaction if antibiotic prescribed^31^ and complaints in relation to parents’ unmet need for antibiotics.^34^ However, studies have also reported parents being satisfied with a thorough examination and reassurance without antibiotic prescription^35,36^ and that patients have good acceptance of a non-antibiotic management plan.^37^

As described in this study, RTIs were the main indication for antibiotics^8,25^ and penicillins were the most commonly prescribed agents in other studies conducted in OOH settings.^6,7,9,11^ During weekdays, most antibiotics were prescribed between 6 and 10pm, when most OOH consultations occur, which has also been described in other OOH studies.^6,7^ This may indicate deficiencies in access to daytime general practice.^38^ OOH doctors prescribing antibiotics for dental complaints may indicate issues with patient access for dental treatment, resulting in patients presenting to OOH doctors’ services where antibiotics are prescribed as an interim measure while awaiting dental treatment.

The proportion of Red antibiotics in the study was considerably lower (19%) compared to the national proportion reported during the study period (35%–45%),^39^ and the national target for primary care in Ireland (34%).^20^ This finding shows sustained improvements from the Cork Kerry region OOH antibiotic quality improvement initiative in 2017–18 with the introduction of an antibiotic trigger tool that reminds prescribers of the preferred Green antibiotics. This initiative was specific to this region, solely in the OOH setting and continues to be used in this OOH service.^17^

Regarding choice of antibiotic, adherence to national guidelines for antibiotic choice for RTIs (77%) was similar to a Dutch study (65%–71%).^11^ The higher proportion of older adults on Red antibiotics in this study highlights an area for AMS intervention. Higher use of broad-spectrum antibiotics in older people has also been described in other studies.^8,40^ Although older adults are more likely to require second- or third-line antibiotics as a result of treatment failure and a higher likelihood of infection with antibiotic-resistant organisms, they are also are more vulnerable to adverse effects, *Clostridioides difficile* infection and drug interactions resulting from polypharmacy. Extra caution should be taken in choice of antibiotic in this population.^41^

This study also shows higher use of Red antibiotics by the overnight shift doctors. One reason for this could be a lower awareness of national antimicrobial guidance in this cohort of locum doctors who do not receive the same level of communication of national key AMS messages as the GPs covering the bright-eye shifts.

Co-amoxiclav, a Red antibiotic, was the second most commonly prescribed antibiotic and accounted for a substantial proportion of antibiotic prescriptions for dental and skin infections (20% and 18%, respectively). It is not a first line antibiotic for most infections in primary care, therefore presents an area of focus for further improvement in antibiotic choice. Encouragingly, quinolone use was very low and they should be restricted to very limited indications due to risk of serious adverse effects.^42^

### Future directions for AMS in OOH services

A key area of focus for AMS highlighted by this study is to reduce antibiotic prescribing antibiotic use for RTIs, particularly in children. Quality indicators for outpatient antibiotic prescribing developed by European Surveillance of Antimicrobial Consumption state that less than 30% consultations for acute bronchitis and less than 20% consultations for tonsillitis, sinusitis and upper RTI in outpatient settings should result in an antibiotic prescription.^43^ Studies have shown a high proportion of RTI consultations for these indications result in an antibiotic prescription in GP consultations in Ireland.^44,45^ Exploring prescribing against these indicators in the OOH services and the factors influencing high rates of antibiotic prescribing for RTIs will highlight specific themes for AMS interventions. The extension of free doctor visits to children under 8 years in August 2023 has the potential to further increase demand on OOH consultations and antibiotic prescribing.

Interventions that have been shown to reduce antibiotic prescribing for RTIs in general practice include communication skills training,^46^ use of patient information leaflets (including for children),^47^ delayed antibiotic strategy^48,49^ and public awareness campaigns.^50^ These also have the potential to reduce antibiotic prescribing in the OOH setting. Some bespoke interventions that have been trialled in OOH setting include using pop-ups on the electronic health record with advice on when to withhold antibiotics and feedback to doctors on prescribing patterns.^51^ These should be considered to complement the antibiotic trigger tool currently used in this OOH service.

As remote consultations resulting in antibiotic prescriptions remain common, AMS in telemedicine will be an important area to address. Some studies have found a higher rate of antibiotic prescribing from remote consultations,^52,53^ which may be related to a higher level of uncertainty with remote consultations leading to more ‘just in case’ antibiotic prescriptions.^54^ One study showed that antibiotic use protocols for telemedicine, supplemented by training and feedback on adherence to guidelines, resulted in a low prescription rate.^55^

C-reactive protein point of care tests have been cited as a useful adjunct for reducing antibiotic prescribing for RTIs. However, three published C-reactive protein point of care test studies conducted in the OOH setting show limited value in reducing antibiotic prescriptions,^33,56,57^ highlighting challenges in training a fluctuant staff group and practicalities of conducting the tests in OOH settings, with inconsistent views of GPs on its utility.^57^

The OOH setting provides a unique opportunity for AMS, as in Ireland most GPs who work in independent practices during the daytime also work in the OOH services. Therefore, AMS initiatives and messaging in the OOH setting are an effective method to capture a significant number of GPs and may in addition influence their daytime practice.

The overnight shift doctors are a unique cohort who do not receive the same level of communication of national key messages on AMS as daytime GPs through the established communication channels, and therefore this cohort may not have the same level of awareness of national guidance and AMS resources as the doctors who also work in daytime general practice. Tailored AMS education for this group of overnight shift doctors should be considered.

### Study limitations

The study examined antibiotic use before and during the COVID-19 pandemic in one region in Ireland, which had previously received some AMS interventions therefore the results may not be generalizable beyond this time period and region. Analysis of antibiotic prescribing in OOH services in other regions in the post pandemic era would be valuable.

More detailed information on all OOH consultations, not just for consultations where antibiotics were prescribed, (e.g. clinical presentation, age and in-person/remote consultation documented), and a skill mix of doctors and case mix of the different shifts, would have been useful to further examine factors that influence antibiotic prescribing to identify more targeted AMS opportunities.

Whether or not patients pay for their OOH consultation would be useful to explore in future studies. There is a substantial fee for doctor consultations for a significant proportion of people in Ireland, including for OOH doctor consultations. A sense of reciprocity to patients may be a driver for increased antibiotic prescribing by doctors particularly for fee-paying patients.^27,58^

Information on whether the antibiotic prescription issued was delayed versus immediate was not available for this study, therefore the use of this strategy could not be examined further. Prescribing patterns at different treatment centres were not examined in this study but may be helpful for local-level interventions at specific treatment centres.

### Conclusion

Antibiotic prescribing rates in OOH settings in this region in Ireland varied considerably over the course of the study period before and during the COVID-19 pandemic. Low antibiotic prescribing levels during the early stages of pandemic were not sustained. Antibiotic prescriptions from remote consultations were common. The use of Red (reserve) antibiotics remains low in this OOH service. A key opportunity for AMS identified is addressing the volume of antibiotic prescribing for RTIs, particularly in children.

## Supplementary Material

dlae009_Supplementary_Data

## Supplementary data

Figure S1 and Tables S1 to S4 are available as Supplementary data at *JAC-AMR* Online.
