# Supplementary material for: Trends in antibiotic prescribing in primary care out-of-hours doctors’ services in Ireland
Source: JAC Antimicrob Resist. 2024 Feb 9;6(1):dlae009. doi: 10.1093/jacamr/dlae009 (PMC10854216; doi:10.1093/jacamr/dlae009)
Supplement: dlae009_Supplementary_Data [file dlae009_supplementary_data.docx]

**Supplementary data**

Table S1: Breakdown for antibiotics and OOH consultations per year per 1000 people for 2020 and 2021.

| Age | Antibiotics prescribed (n) 2020 | Antibiotics prescribed (n) 2021 | Antibiotics prescribed (n) study total | % age category prescribed antibiotics / all antibiotics prescribed  2020 | % age category prescribed antibiotics / all antibiotics prescribed  2021 | % age category prescribed antibiotics / all antibiotics prescribed  2020+2021 | Cork Kerry population census  2016 | Number of antibiotics prescribed per 1000 people 2020 | Number of antibiotics prescribed per 1000 people  2021 |
| --- | --- | --- | --- | --- | --- | --- | --- | --- | --- |
| 0-6 yrs | 4369 | 7609 | 14148 | 17% | 22% | 20% | 67283 | **65** | **113** |
| 7-16yrs | 3460 | 3317 | 8901 | 13% | 10% | 13% | NI^¥^* | NI^¥^ | NI^¥^ |
| 17-64 yrs | 13621 | 17758 | 35005 | 53% | 52% | 51% | NI^¥^ | NI^¥^ | NI^¥^ |
| 65+ yrs | 4220 | 5416 | 10714 | 16% | 16% | 16% | 98857 | **43** | **55** |
| total | 25670 | 34349 | 69017 | - | - | - | 690575 | **37** | **50** |

^¥^NI, Not investigated

Number of OOH consultations per 1000 people 2020: 179,889/690,575 x 1000 = 260 consultations per 1000 people

Number of OOH consultations per 1000 people 2021: 197,433 / 690575 x 1000 = 286 consultations per 1000 people

Table S2: Indication for antibiotic stratified by age

|  | | | Indication | | | | | | | | Total |
| --- | --- | --- | --- | --- | --- | --- | --- | --- | --- | --- | --- |
|  |  |  | Dental | GI | OBGY | RTI | SSTI | STI | Unknown | UTI |  |
| Age Category | 0-6 yrs | *n* | 63 | 12 | 5 | 9921 | 586 | 51 | 2847 | 663 | 14148 |
|  |  | *% within Age Category* | 0.4% | 0.1% | 0.0% | 70.1% | 4.1% | 0.4% | 20.1% | 4.7% | 100.0% |
|  | 7-16 yrs | *n* | 212 | 12 | 6 | 5166 | 910 | 17 | 1951 | 627 | 8901 |
|  |  | *% within Age Category* | 2.4% | 0.1% | 0.1% | 58.0% | 10.2% | 0.2% | 21.9% | 7.0% | 100.0% |
|  | 17-64 yrs | *n* | 1618 | 280 | 128 | 12858 | 5884 | 157 | 8152 | 5928 | 35005 |
|  |  | *% within Age Category* | 4.6% | 0.8% | 0.4% | 36.7% | 16.8% | 0.4% | 23.3% | 16.9% | 100.0% |
|  | 65+ yrs | *n* | 108 | 136 | 5 | 3055 | 1280 | 13 | 2795 | 3322 | 10714 |
|  |  | *% within Age Category* | 1.0% | 1.3% | 0.0% | 28.5% | 11.9% | 0.1% | 26.1% | 31.0% | 100.0% |
| Total | | *n* | 2001 | 440 | 144 | 31000 | 8660 | 238 | 15745 | 10540 | 68768 |
|  |  | *% within Age Category* | 2.9% | 0.6% | 0.2% | 45.1% | 12.6% | 0.3% | 22.9% | 15.3% | 100.0% |

Abbreviations: GI = gastrointestinal / intra-abdominal infection; RTI = respiratory tract infection; OBGY = gynaecological and pregnancy/postpartum infection;

SSTI = skin and soft tissue infection; STI = sexually transmitted infection; UTI = urinary tract infection.

Table S3: Indication for antibiotic stratified by gender

|  | | | Indication | | | | | | | | Total |
| --- | --- | --- | --- | --- | --- | --- | --- | --- | --- | --- | --- |
|  |  |  | Dental | GI | OBGY | RTI | SSTI | STI | Unknown | UTI |  |
| Patient Gender | Female | *Count* | 1092 | 282 | 142 | 17205 | 4653 | 13 | 9223 | 8614 | 41224 |
|  |  | *% within Indication* | 54.6% | 63.9% | 98.6% | 55.2% | 53.7% | 5.5% | 58.4% | 81.7% | 59.7% |
|  | Male | *Count* | 909 | 159 | 2 | 13969 | 4014 | 225 | 6583 | 1932 | 27793 |
|  |  | *% within Indication* | 45.4% | 36.1% | 1.4% | 44.8% | 46.3% | 94.5% | 41.6% | 18.3% | 40.3% |
| Total | | *Count* | 2001 | 441 | 144 | 31174 | 8667 | 238 | 15806 | 10546 | 69017 |

Abbreviations: GI = gastrointestinal / intra-abdominal infection; RTI = respiratory tract infection; OBGY = gynaecological and pregnancy/postpartum infection;

SSTI = skin and soft tissue infection; STI = sexually transmitted infection; UTI = urinary tract infection

Table S4: Number and proportion of different antibiotics prescribed over the study period

| **Antibiotic** | **Number of prescriptions** | **% of total antibiotic prescriptions** |
| --- | --- | --- |
| Amoxicillin | 27409 | 39.7 |
| Co-amoxiclav | 8495 | 12.3 |
| Flucloxacillin | 7120 | 10.3 |
| Cefalexin | 6530 | 9.5 |
| Phenoxymethylpenicillin | 4566 | 6.6 |
| Doxycycline | 3440 | 5.0 |
| Clarithromycin | 3208 | 4.6 |
| Nitrofurantoin | 2505 | 3.6 |
| Trimethoprim | 2148 | 3.1 |
| Other cephalosporins | 1622 | 2.4 |
| Ciprofloxacin | 846 | 1.2 |
| Metronidazole | 451 | 0.7 |
| Azithromycin | 259 | 0.4 |
| Fosfomycin | 165 | 0.2 |
| Erythromycin | 111 | 0.2 |
| Clindamycin | 88 | 0.1 |
| Ofloxacin | 26 | 0.04 |
| Moxifloxacin | 14 | 0.02 |
| Lymecycline | 10 | 0.01 |
| Levofloxacin | 4 | 0.006 |
| Total | 69017 | 100.0 |


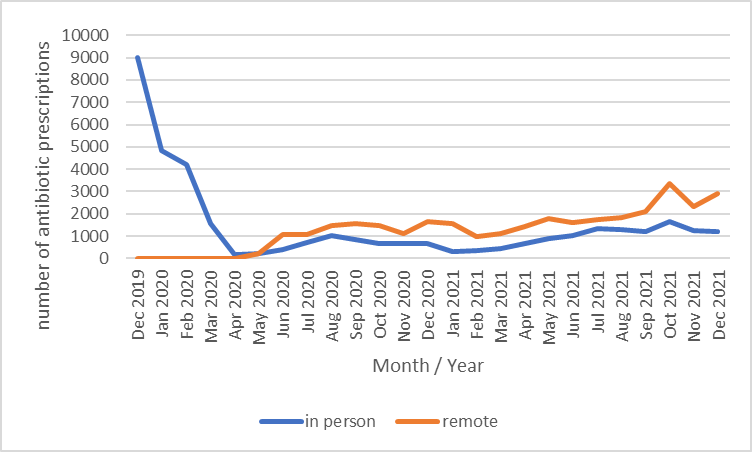


Figure S1: Number of antibiotic prescriptions in remote versus in person consultations over the study period.
